# Microwave Ablation in the Proximity of Surgical Clips: Is there a Safety Issue?

**DOI:** 10.1007/s00270-020-02453-1

**Published:** 2020-03-31

**Authors:** Martin Liebl, Maximilian Schulze-Hagen, Markus Zimmermann, Federico Pedersoli, Christiane Kuhl, Philipp Bruners, Peter Isfort

**Affiliations:** 1 Department of Diagnostic and Interventional Radiology, University Hospital RWTH Aachen, Pauwelsstraße 30, 52074 Aachen, Germany; 2 Hôpital Kirchberg (Hôpitaux Robert Schuman), 9, Rue Edward Steichen, 2540 Luxembourg, Luxembourg

**Keywords:** Microwave ablation, MWA, Surgical clips, Metal, Liver, Heating, Thermal damage

## Abstract

**Purpose:**

The purpose of this study was to evaluate the heat generation of surgical clips within the target area of MWA and the influences on the ablation volume.

**Materials and Methods:**

In bovine liver tissue, 42 ex vivo microwave ablations (60 W; 180 s) were performed. During ablation, the temperature was measured continuously at 4 points of interest (POI), in a distance of 7.5 and 15 mm on each side of the microwave antenna, with a titanium surgical placed at one 7.5-mm POI. Ablation volumes containing large vessels (*n* = 10) were excluded. For every POI, the mean temperature of 32 ablations was calculated. The mean temperatures were compared between the 4 POI and statistically analyzed using the Student’s *t* test.

**Results:**

The mean maximum temperatures at the side of the clip were 88.76 °C/ 195 s and 52.97 °C/ 195 s and at the side without clip 78.75 °C/ 195 s and 43.16 °C/ 195 s, respectively, at POI 7.5 mm and POI 15 mm. The maximum difference of mean temperatures for POI 7.5 mm was 12.91 °C at 84 s (*p* = 0.022) and for POI 15 mm 9.77 °C at 195 s (*p* = 0.009). No significant changes in size and shape of the ablation zone could be determined.

**Conclusions:**

Our study demonstrated significantly higher temperatures adjacent to surgical clips. Also, the temperatures distal to the titanium clip were higher compared to the control location without clip. These findings suggest an increased risk of thermal damage to surrounding tissues during MWA, especially in case of immediate contact to surgical clips.

## Purpose

A broad spectrum of treatment options is available for the treatment of primary and secondary hepatic malignancies. Depending on the tumor entity, location and size, different treatment strategies are preferable. Profound improvements in the outcomes of patients with metastatic colorectal cancer (CRC) as well as selected other metastases have been achieved by means of increasingly extensive hepatic resection and more effective systemic therapy [1]. Additionally more extensive surgery in patients suffering from hepatocellular carcinoma (HCC) results in good oncological results [2].

Nevertheless, recurrence of liver metastases after liver resection is common, reported to be as high as 48% of patients after resection of CRC liver metastases [3]. In case of local recurrence repeated liver resection is often not feasible due to a limited liver volume and an increased surgical risk [4]. On the other hand, local ablative techniques can provide excellent tumor control with high preservation of healthy liver tissue. Microwave ablation (MWA) is a powerful alternative to radiofrequency ablation (RFA) with several advantages: MWA is independent of the surrounding tissue impedance, achieving higher temperatures and thus creating larger ablation volumes in a shorter time [5, 6]. Furthermore, MWA is less prone to heat sinks of adjacent larger blood vessels [7–9]. These advantages of MWA are also its flaws, as the higher thermal efficacy of MWA comes along with an increased risk of injury to adjacent critical tissues.

However, local tumor ablation adjacent to former resection sites bares another, perhaps underestimated risk: Surgical clips at the resection margin could generate unwanted heating effects caused by the energy deposition during MWA. Heating of surgical clips may cause severe burns to adjacent organs that are commonly located in immediate contact with the resection margin, like bowel, stomach, pancreas, spleen or the kidneys. Therefore, the purpose of this study was to evaluate the heat generation of surgical clips within the target area of MWA, as well as possible influences on the ablation volume.

## Methods

**Fig. 1:**
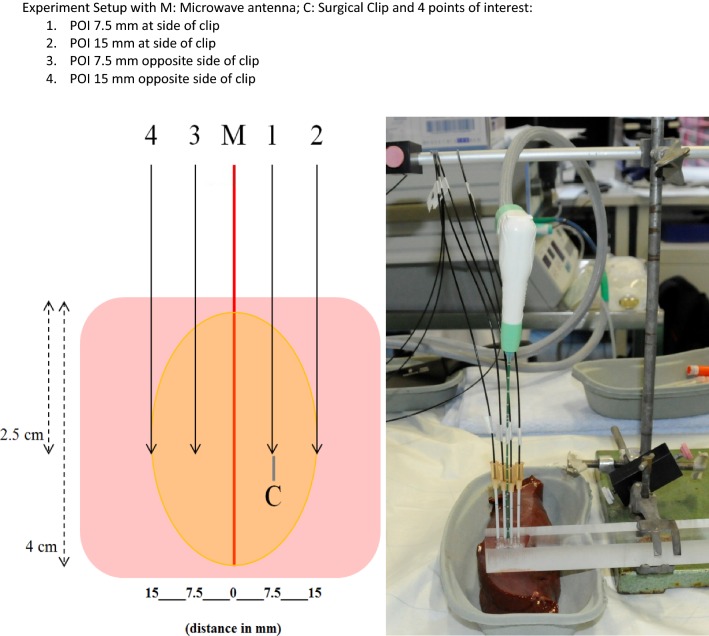
Experimental setup with M: microwave antenna; C: surgical clip and 4 points of interest: 1. POI 7.5 mm at side of clip. 2. POI 15 mm at side of clip. 3. POI 7.5 mm opposite side of clip. 4. POI 15 mm opposite side of clip

In an ex vivo setting, a total of 42 ablations was performed with a microwave antenna (AMICA ®—AGN-3.0 Generator working with 2450 MHz and 16G/20 cm probe; Mermaid Medical, Stenløse, Denmark) inserted into freshly excised bovine liver tissue. In parallel fashion 4 temperature probes were inserted into the target area of the ablation. An acrylic glass spacer was used to ensure parallel guidance of the MWA antenna as well as the temperature probes on a straight line with defined distances of 7.5 mm between antenna and temperature probes as well as between temperature probes. The microwave antenna was inserted 4 cm into the liver tissue, and the 4 temperature probes were inserted 2.5 cm, to place the measuring tip in the middle of the long axis of the active tip (Fig. 1). A standardized ablation protocol with an energy application of 60 Watts for 3 min was applied. Temperature measurements were taken using a fluoroptic thermometer and 4 fiber optic temperature probes (FOT Lab Kit four-channel Fluoroptic® thermometry; Luxtron Fluoroptic® probes; Luxtron Corporation, Santa Clara, CA). One microwave antenna and therewith two points of interest (POI) on each side of the MWA antenna are used for temperature monitoring: one at 7.5 mm (POI 7.5 mm) and one at 15 mm (POI 15 mm) distance to the microwave antenna. At one side of the antenna, a titanium surgical clip (Premium Surgiclip™ II clip applier, size Medium; Covidien/Medtronic Minneapolis, Minnesota) was placed in direct contact with POI 7.5 mm. Temperatures were measured every second at all 4 POI, starting 15 s before microwave ablation was started. Temperature measurements were documented using Tera Term Pro software (Tera Term Project, version 2.3 update 4.85) on a windows-based computer. All experiments were performed at room temperature (~ 21 °C).

After the experiments, the liver specimens were dissected parallel to the long axis of the microwave antenna along the straight line with the temperature probes. Homogeneity of the liver tissue was evaluated visually, and ablation volumes containing large vessels (*n* = 10) were excluded.

Of the remaining 32 treated liver specimens, ablation zone sizes were measured on both sides of the microwave antenna (short axis) and along the antenna shaft (long axis).

Statistical analyses.

Overall ablation volumes could be calculated using the ellipsoid formula $$\frac{4}{3}$$$$\pi$$ x length x width x height.

For every POI, the mean temperature of 32 measurements at each time point was calculated.

The differences in mean temperatures were compared between the 4 POI and statistically analyzed using the Student’s *t* test (PASW version 18, SPSS Inc., Hong Kong).

## Results

The mean maximum temperatures of 32 measurements for every POI were reached at 195 s: 88.76 °C (min: 24.3 °C /max: 107.1 °C /SD 25.6 °C) and 52.97 °C (min: 24.1 °C /max: 88.2 °C /SD 16.7 °C), respectively, at POI 7.5 mm and POI 15 mm at the side with clip and 78.75 °C (min: 29.7 °C /max: 103.1 °C /SD 19.1 °C) and 43.16 °C (min: 23.2 °C /max: 87.0 °C /SD 13.9 °C), respectively, at POI 7.5 mm and POI 15 mm at the side without clip.

**Fig. 2:**
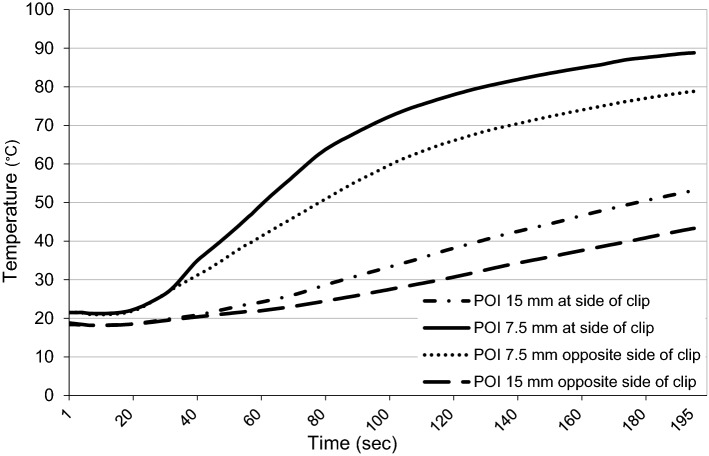
Mean temperature curve for 4 POIs. Total acquisition time 195 s. Start of the ablation 15 s after start of temperature measurements with a total time of ablation of 180 s

The maximum difference of mean temperatures between the side of the clip and the side without clip for POI 7.5 mm was 12.91 °C at 84 s (*p* = 0.022). The maximum difference of mean temperatures for POI 15 mm was 9.77 °C at the end of the ablation at 195 s (*p* = 0.009). See Fig. 2 for temperature curves.

**Fig. 3:**
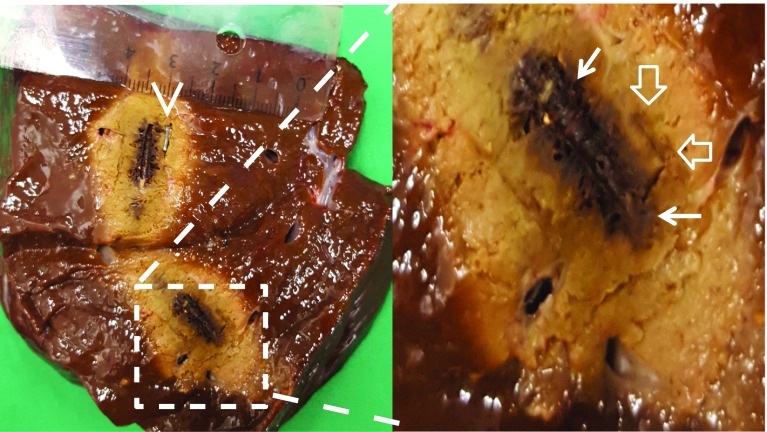
Macroscopic photograph of liver specimen cut along the axis of temperature probes and microwave antenna. Arrowhead: surgical clip in MWA target area. Arrows: charring of ablated liver tissue around the tip of the microwave antenna. Open arrows: A small amount of tissue charring can be seen at the location of the surgical clip

Visual examination of the ablation zones revealed minimal charring of the liver tissue adjacent to the surgical clip in 4 of 32 ablations (Fig. 3). No significant changes in size and shape of the ablation zone could be determined.

## Discussion

The aim of our study was the evaluation of possible unwanted heating effects caused by surgical clips within the ablation zone of MWA in liver tissue. Our results show significant additional heating effects at the site of a surgical clip during MWA. Furthermore, critical temperatures above 60 degrees Celsius are reached sooner adjacent to the clip, aggravating the risk of thermal damage to adjacent organs at the liver resection margin.

Although repeated hepatic resection has proven to provide prolonged survival in this scenario, it is technically challenging and associated with increased morbidity and mortality [10]. Postoperative adhesions, scaring and distorted anatomy of the remnant liver pose major challenges, even for experienced surgeons. Additionally, patients with technically resectable lesions may not have adequate hepatic reserve to undergo surgery [11]. In these cases, local ablative techniques, such as radiofrequency ablation and microwave ablation, may be suitable treatment options with lower morbidity and mortality compared to both laparoscopic and open liver surgery [12]. Furthermore, in contrast to repetition of surgery, ablation does not stand in the way of possible future resection.

Both in RFA and MWA tissue heating is induced by electromagnetic waves. When good electric conductors, such as metals, are placed within the ablation zone, extensive heating may occur due to ohmic heating. A study of Cardinal et al. showed significantly more heating within the area of RFA with injected gold nanoparticles compared to a control with water injection [13]. Also bigger metallic medical devices, such as defibrillator, leads or circular mapping catheters, potentiated the effects of RFA in bovine myocardium [14]. However, small metallic implants showed none or lesser thermal effects. Lin et al. studied the influences of silver clips and 125-I seeds within the target area of RFA, without significant thermal differences to the control group [15]. Even closer to our study design, Boll et al. investigated the effect of titanium surgical clips in an ex vivo porcine liver model. No aberrant conduction was observed for clips located 20 mm and further from the radiofrequency probe, and the authors concluded that RFA can be safely performed in patients with implanted titanium surgical clips [16]. However, the report shows faster heat generation around titanium clips within the ablation zone, strongly suggesting the presence of ohmic heating; however, no additional safety concerns were stated. To our knowledge, no studies exist on the effect of titanium surgical clips under MWA, although the energy deposition clearly exceeds these applied in RFA.

“Don't put metal objects in your microwave oven” is a commonly known advice. It is proven by physics that certain metal objects can generate significant heating effects when microwaves are applied. Vollmer et al. described various microwave experiments, where thin metal wires rapidly heat up by several hundred degrees within seconds [17]. Medical high-power MWA is considered to generate larger and more rounded volumes of necrosis with minimization of the heat-sink effect compared to RFA [18]. MWA of tumor recurrence adjacent to resection sites containing metal surgical clips could cause a so-far underestimated problem: unwanted heating effects of metal clips within the ablation zone. Consequently, vital organs adjacent to the clips of the resection margin, such as the kidney, stomach or colon, could be at risk of severe thermal damage.

**Fig. 4:**
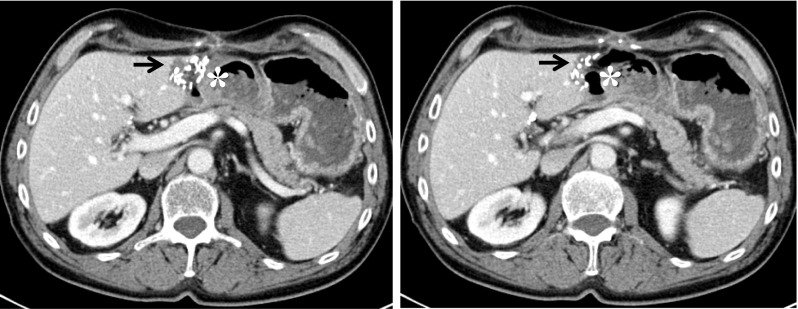
Two subsequent CT images of a patient with tumor recurrence at the left hepatic resection margin (arrows). The air-filled colon transversum lies directly adjacent to the clips of the resection margin (asterisk)

Our study showed a significant increase in local temperature at the site of a surgical clip within the target area of a MWA, when compared to the same distance from the microwave antenna without clip. The mean temperature difference between POI 7.5 mm at the side of the clip compared to the same POI on the opposite side was 12.91 degrees Celsius toward the end of a 3-min ablation protocol. Furthermore, the mean temperature at POI 15 mm on the side of the clip was 9.77 degrees higher compared to the same POI on the opposite side. Thus, substantial heating effects occur at the site of surgical clips and these are propagated into the surrounding tissue. As commonly known from RFA, the range of direct thermal conduction within liver tissue appears to be rather small, as no significant changes in size and shape of the ablation zones were found. Nevertheless, CT scans of patients after liver resection frequently show immediate contact of the resection margin (with surgical clips) to surrounding organs, as demonstrated in Fig. 4. As a consequence, surgical clips are in direct contact to vulnerable structures such as the kidney or bowel without any thermal insulation in-between.

The amount of thermal tissue damage depends on two variables, the temperature applied and the exposure time. From 42 to 60 degrees Celsius, the time that is required to achieve irreversible tissue damage decreases exponentially, with rapid protein denaturation and coagulative necrosis above 60 degrees Celsius [19, 20]. The results of our experiments show not only a higher mean temperature adjacent to surgical clips at the end of the ablation. The critical temperature of 60 degrees Celsius is reached 26 s faster at the site of the clip and thus will be applied longer within the ablation time, as demonstrated in Fig. 2. As a result, more excessive thermal damage must be expected. In 4 of the 32 ablations, these findings are confirmed by a small amount of charring of the liver tissue adjacent to the surgical clip (Fig. 3).

The results of our study were limited by several factors. Although MWA is less susceptible to the cooling effects of vessels than RFA [7], this study in ex vivo bovine liver was limited by the absence of blood flow. The higher water content of many tumor tissues and therefore a potentially higher susceptibility to energy depositions by MWA could not be taken into account, as only healthy bovine tissue was used in our study [21–23]. Perhaps for that reason we did not find changes in ablation size and shape due to temperature differences. Furthermore, potential synergistic thermal effects of multiple surgical clips and different configurations of clips were not investigated. We tested common surgical clip material used by surgeons of our hospital for liver resection. We did not investigate different kinds of material in this study.

## Conclusions

In conclusion, our study demonstrated significantly higher temperatures adjacent to surgical clips within the target area of a MWA, compared to a control location at the same distance to the antenna without clip. These findings suggest that there is an increased risk of thermal damage of tissue adjacent to surgical clips during MWA. Therefore, protective measures, like local gas or hydrodissection, have to be considered to safely perform MWA in cases of local recurrence after liver resection.

## Abbreviations

CRC: Colorectal cancer
HCC: Hepatocellular carcinoma
MWA: Microwave ablation
RFA: Radiofrequency ablation
POI: Point of interest
